# Histotripsy ablation for the treatment of feline injection site sarcomas: a first-in-cat *in vivo* feasibility study

**DOI:** 10.1080/02656736.2023.2210272

**Published:** 2023

**Authors:** Lauren Ruger, Ester Yang, Sheryl Coutermarsh-Ott, Elliana Vickers, Jessica Gannon, Marlie Nightengale, Andy Hsueh, Brittany Ciepluch, Nikolaos Dervisis, Eli Vlaisavljevich, Shawna Klahn

**Affiliations:** a Department of Biomedical Engineering and Mechanics, VA Polytechnic Institute and State University, Blacksburg, VA, USA; b Department of Small Animal Clinical Sciences, Virginia-MD College of Veterinary Medicine, Blacksburg, VA, USA; c Virginia Tech Animal Cancer Care and Research Center, Virginia-Maryland College of Veterinary Medicine, Roanoke, VA, USA; d Biomedical Sciences and Pathobiology, Virginia Polytechnic Institute and State University, Blacksburg, VA, USA; e Graduate Program in Translational Biology, Medicine and Health, Virginia Polytechnic Institute and State University, Roanoke, VA, USA; f Department of Internal Medicine, Virginia Tech Carilion School of Medicine, Roanoke, VA, USA

**Keywords:** Soft tissue sarcoma, feline, histotripsy, focused ultrasound, cancer, ablation, immuno-oncology

## Abstract

**Purpose:**

Feline soft tissue sarcoma (STS) and injection site sarcoma (fISS) are rapidly growing tumors with low metastatic potential, but locally aggressive behavior. Histotripsy is a non-invasive focused ultrasound therapy using controlled acoustic cavitation to mechanically disintegrate tissue. In this study, we investigated the *in vivo* safety and feasibility of histotripsy to treat fISS using a custom 1 MHz transducer.

**Materials and Methods:**

Three cats with naturally-occurring STS were treated with histotripsy before surgical removal of the tumor 3 to 6 days later. Gross and histological analyses were used to characterize the ablation efficacy of the treatment, and routine immunohistochemistry and batched cytokine analysis were used to investigate the acute immunological effects of histotripsy.

**Results:**

Results showed that histotripsy ablation was achievable and well-tolerated in all three cats. Precise cavitation bubble clouds were generated in all patients, and hematoxylin & eosin stained tissues revealed ablative damage in targeted regions. Immunohistochemical results identified an increase in IBA-1 positive cells in treated tissues, and no significant changes in cytokine concentrations were identified post-treatment.

**Conclusions:**

Overall, the results of this study demonstrate the safety and feasibility of histotripsy to target and ablate superficial feline STS and fISS tumors and guide the clinical development of histotripsy devices for this application.

## Introduction

Feline soft tissue sarcomas (STS), including feline injection site sarcomas (fISS), are rapidly growing tumors with low metastatic potential, but locally aggressive behavior [1]. Currently, the combination of surgery and radiation therapy is recommended to reduce the chances of local recurrence [2]. Unfortunately, many tumors cannot be completely resected at the time of diagnosis due to delayed identification, diagnosis, and/or the location of the mass, resulting in ~28% to 45% of tumors recurring after multimodality treatment with surgery and RT [2-4]. As a result, there is a critical need for novel, less invasive, and more effective therapies to treat feline STS and fISS.

High-intensity focused ultrasound (HIFU) is a noninvasive ablation technique with the ability to destroy tissue by thermal or mechanical means. While traditional HIFU relies on the absorption of ultrasound energy to thermally destroy tissue, histotripsy is a precision non-thermal focused ultrasound therapy that mechanically breaks down tissue without inducing thermal coagulation [5-7]. Histotripsy utilizes short, high-amplitude ultrasound pulses to generate acoustic cavitation bubble clouds within a target tissue; the bubble cloud then expands and contracts, disintegrating the tissue into its sub-cellular components [5,8]. Histotripsy treatment is typically guided in real-time by ultrasound imaging and can target a treatment area with millimeter precision, with only the region exceeding the tissue’s intrinsic threshold subject to cavitation damage [9-12]. Histotripsy also offers tissue selectivity and can spare nearby critical structures, such as large blood vessels, nerves, and bone, due to their increased mechanical strength while still completely emulsifying the target tissue [13-15].

Thermal HIFU has been investigated for a number of applications in humans, including the ablation of solid tumors, but remains limited by the risks of damaging neurovascular tissues, thermal conduction to adjacent tissues causing off-target damage, the heat-sink effect, and/or the possibility of skin burns [16]. In contrast, previous studies investigating histotripsy for a variety of applications have shown that histotripsy does not induce significant changes in temperature on overlying tissues or at the treatment focus, minimizing those risks [7,17,18]. Recently, our team has completed two veterinary clinical trials investigating thermal HIFU and histotripsy for the ablation of STS in dogs with spontaneous tumors, but to date, no studies assessing histotripsy feasibility for feline cancer have been conducted [19,21,22). In both canine studies, predictable ablation of the target volume was achieved with a clear demarcation histologically between treated and untreated tissue. Thermal HIFU and histotripsy treatments alike were well-tolerated by the canine patients, but increased adverse events were noted for the HIFU treatments, including second- and third-degree skin burns [19]. Feline STS are often smaller in size than their canine counterparts, with a larger percentage of the tumor volume located immediately beneath the skin’s surface. These characteristics make the burn risk particularly concerning for feline STS, suggesting that histotripsy may be the preferred ablation method for this application.

In addition to its ablative potential, recent work suggests that histotripsy may also induce immune activation toward an anti-tumor immune response [20-23]. The acellular debris left behind after histotripsy ablation may contain tumor-associated antigens and/or damage-associated molecular patterns (DAMPs), which may serve to activate the immune system to recognize and eliminate tumors cells. The anti-tumor immune response may then stimulate local and systemic immune responses [24]. Spontaneous tumors in companion animals are increasingly recognized for their value in translational oncology research and share many similarities with human cancers, offering a unique opportunity to evaluate and refine immunotherapy strategies [25,26]. Immunological changes after thermal HIFU and histotripsy ablation for canine STS were observed in our previous work [19,21,22]. Following thermal HIFU ablations, 28 genes were identified with statistically significant differential expressions and increases in IBA-1 positive cells were noted at the treatment interface [19]; following histotripsy ablation, 79 genes were significantly upregulated and increases in the IBA-1/CD206 double positive cell population in the treated regions of the tumors were observed [21,222]. Significant work remains to fully characterize the immunological impact of thermal HIFU and histotripsy ablations, particularly between species and tumor subtypes.

In this pilot study, we investigated the *in vivo* safety and feasibility of ablating feline STS/fISS in cats with spontaneous tumors. Secondarily, we characterized the impact of histotripsy ablation on the acute immunologic response both locally (i.e., within the tumor microenvironment) and systemically. A custom 1 MHz histotripsy system was used to target portions of solid tumors in three client-owned cats with suspected injection site sarcomas, followed by surgical resection of the tumor three to six days after histotripsy treatment. Gross and histopathologic assessments were conducted to determine the efficacy of the histotripsy ablation, and immunohistochemical and serum cytokine analyses were completed to investigate the impact of a single, partial histotripsy treatment on the immunologic response for feline STS.

## Methods

### Patient screening and enrollment

Client-owned cats with naturally-occurring peripheral soft tissue tumors were screened at the Virginia-Maryland College of Veterinary Medicine Animal Cancer Care and Research Center (ACCRC) over a 2-month period (22 September 2021 – 18 November 2021) and enrolled in a prospective, singlearm, pilot study of histotripsy treatment in cats diagnosed with peripheral soft tissue sarcoma. Feline patients with a cytologic or histologic diagnosis of peripheral, malignant soft tissue sarcoma were considered for enrollment. Study inclusion criteria included tumor diameter of ≥ 2 cm in the longest dimension, complete tumor resectability as determined by an American College of Veterinary Surgeons (ACVS)-board-certified veterinary surgeon, expected patient survival of > 4 weeks without treatment, and compliance with all scheduled treatment visits. At the time of study screening, patients were required to undergo routine laboratory blood-work and thoracic and abdominal imaging. Patients were excluded if the tumor was non-resectable or if the recommended surgical resection was declined; if the patient had definitive therapy other than surgery within the past 3 weeks; or if they had a significant co-morbidity (significant cardiac, pulmonary, or renal dysfunction, ALT or AST values ≥ 3x the upper reference limit). The owners of eligible cats were offered standard treatment options, including palliative care, and informed consent was obtained for all enrolled cats. This study was approved by the University Institutional Animal Care and Use Committee (IACUC protocol #20-180) and the College of Veterinary Medicine Hospital Board.

### Evaluation timeline

**Figure 1.**
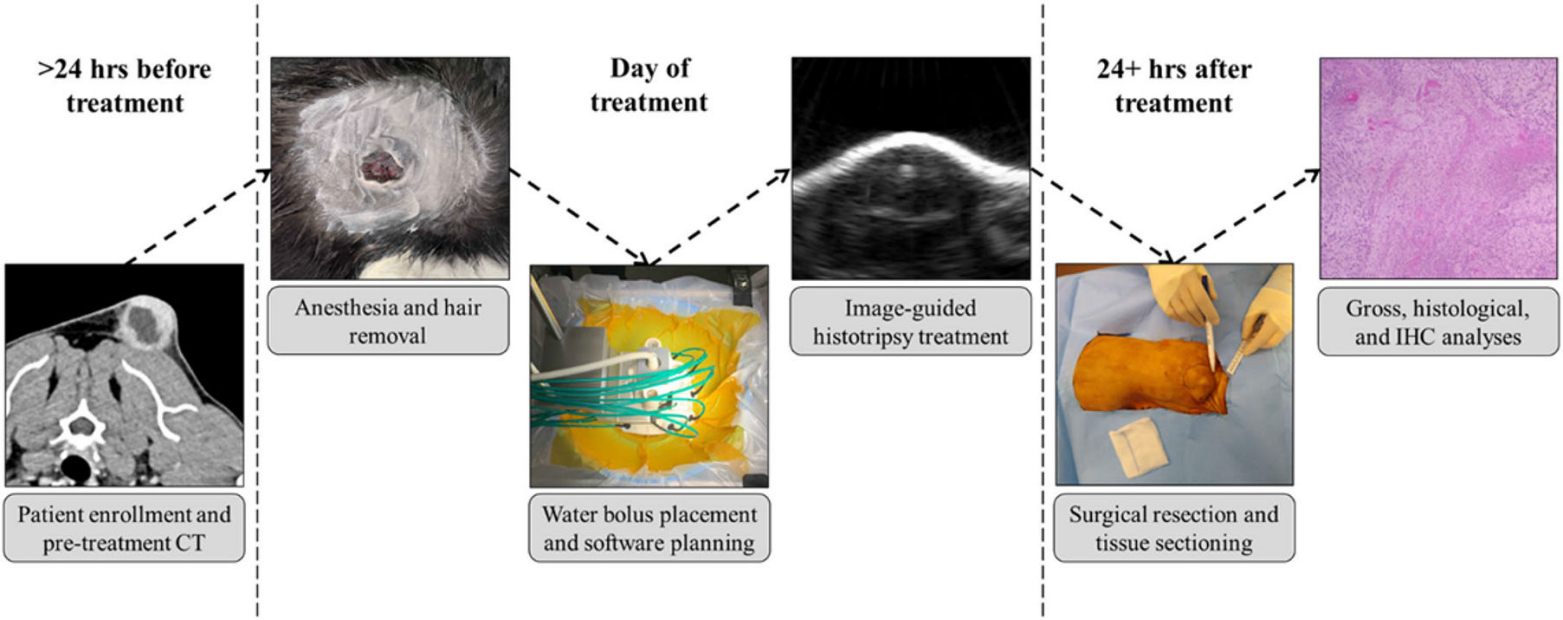
Study workflow and timeline. After patient screening and treatment planning, fur overlying the treatment zone was removed and patients were anesthetized. Then, an automated histotripsy treatment was completed with real-time ultrasound image guidance. Tumors were surgically resected 3 to 6 days post-treatment, and additional analyses were completed to assess the completeness of histotripsy ablation and immunological changes following histotripsy. Whole blood was drawn at three points throughout the study (pretreatment, 1 day post-treatment, and immediately prior to surgical resection) for batched cytokine analysis.

Within 10 days prior to histotripsy treatment, the baseline evaluation for each patient included a physical examination, complete blood count (CBC) and serum biochemistry analyses, caliper measurements and gross photographs of the tumor, and a contrast-enhanced computed tomography (CT) scan (SOMATOM Confidence^®^ RT) of the thorax, abdomen, and tumor. Also prior to histotripsy, pretreatment biopsies were collected from tumor regions outside of the planned treatment zone. Two patients (Patient #1 and Patient #2) had their biopsies collected five days prior to histotripsy treatment, while one patient biopsy (Patient #3) was completed immediately prior to histotripsy. On the day of histotripsy treatment, a physical exam was completed to assess for any changes from the baseline visit, and tumor photographs were obtained immediately prior to and immediately following treatment. One day post-treatment, patients underwent physical exams, and tumor measurements/photographs were collected. Three to six days post-treatment, tumor measurements and photographs were collected, and CBC and serum biochemistry analyses were completed immediately prior to surgical resection by a Diplomate of the ACVS and Fellow in Surgical Oncology. After surgery, patients were recovered in the intensive care unit and discharged to the care of their owners when deemed appropriate by the attending clinician. Two weeks after surgery, patients were examined and the surgery site was photographed at a final study recheck visit. Post-study monitoring recommendations were made as appropriate by the study clinician depending on the patient’s tumor type and stage. Patient outcome was continually monitored upon completion of the study through follow-up visits and/or communication with the owner or primary care veterinarian. The study workflow and timeline are summarized in Figure 1.

### Histotripsy system and calibration

**Figure 2.**
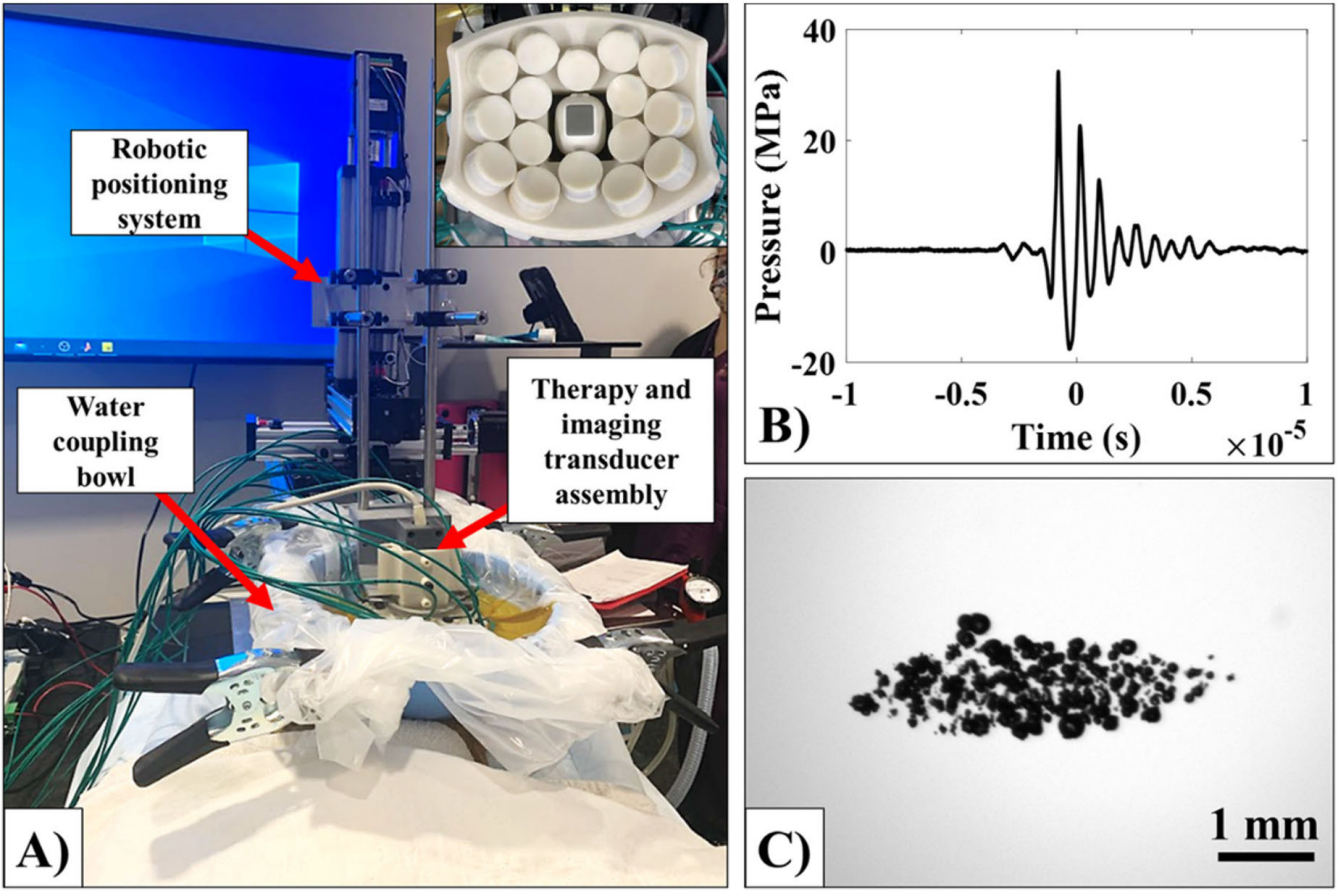
Experimental histotripsy set-up and transducer characterization. (A) A 1-MHz histotripsy transducer with a coaxially aligned imaging probe (insert) was used to treat all patients. The histotripsy system consisted of a robotic positioner supporting the therapy and imaging transducer assembly and a water coupling bowl. (B-C) Before treatment, the histotripsy transducer was characterized. (B) A representative pressure waveform from the 1 MHz transducer used in this study collected at ~−17.5 MPa peak negative pressure. (C) Bubble cloud dimensions were measured to determine volumetric treatment spacing. A representative bubble cloud at ~−34 MPa peak negative pressure is shown.

In this study, a custom 16-element, 1 MHz histotripsy transducer with a geometric focus of 58 mm, maximal elevational and transverse aperture sizes of 66.7 mm and 82.7 mm, respectively, and corresponding f-numbers of 0.87 (elevational) and 0.70 (transverse) was used. The array transducer was built in-house using rapid prototyping technology and integrated onto a computer-guided 3-D robotic positioner before treatment (Figure 2(A)). A phased array ultrasound probe with a frequency range of 3 – 8 MHz (P8-3L10SI-6, Telemed, Vilnius, Lithuania) was coaxially aligned within the transducer for treatment guidance and monitoring. The transducer was driven using a high-voltage pulser designed to generate single-cycle therapy pulses, controlled by a pre-programmed field-programmable gate array (FPGA) board (Altera DE0-Nano Terasic Technology, Dover, DE, USA) and a custom MATLAB script (The MathWorks, Natick, MA, USA), and powered by a high voltage DC power supply (GENH750W, TDK-Lambda, National City, CA, USA).

Before treatment, focal pressure waveforms for the histotripsy transducer (Figure 2(B)) were measured in degassed water using a high-sensitivity reference rod hydrophone (HNR-0500, Onda Corp., Sunnyvale, CA, USA) and a cross-calibrated custom-built fiber optic probe hydrophone (FOPH) aligned at the transducer’s focus as previously described [27,28]. Briefly, the rod hydrophone was used to measure the lateral, elevational, and axial 1-D beam profiles of the transducer at a peak negative pressure (*p*−) of ~1.5 MPa. The measured full-width half-maximum (FWHM) dimensions for the 1 MHz transducer were 0.63 mm, 1.2 mm, and 4.8 mm in the transverse, elevational, and axial directions, respectively. Then, focal pressures were measured directly with the FOPH up to a peak negative pressure of ~20 MPa. At peak negative pressures greater than 20 MPa, the focal pressure was estimated by summing measurements from half and quarter subsets of the elements to prevent cavitation from forming on the fiber [29]. All waveforms were measured using a Tektronix TBS2000 series oscilloscope at a sample rate of 500MS/s, averaged over 128 pulses, and recorded in MATLAB.

### Histotripsy treatment

**Figure 3.**
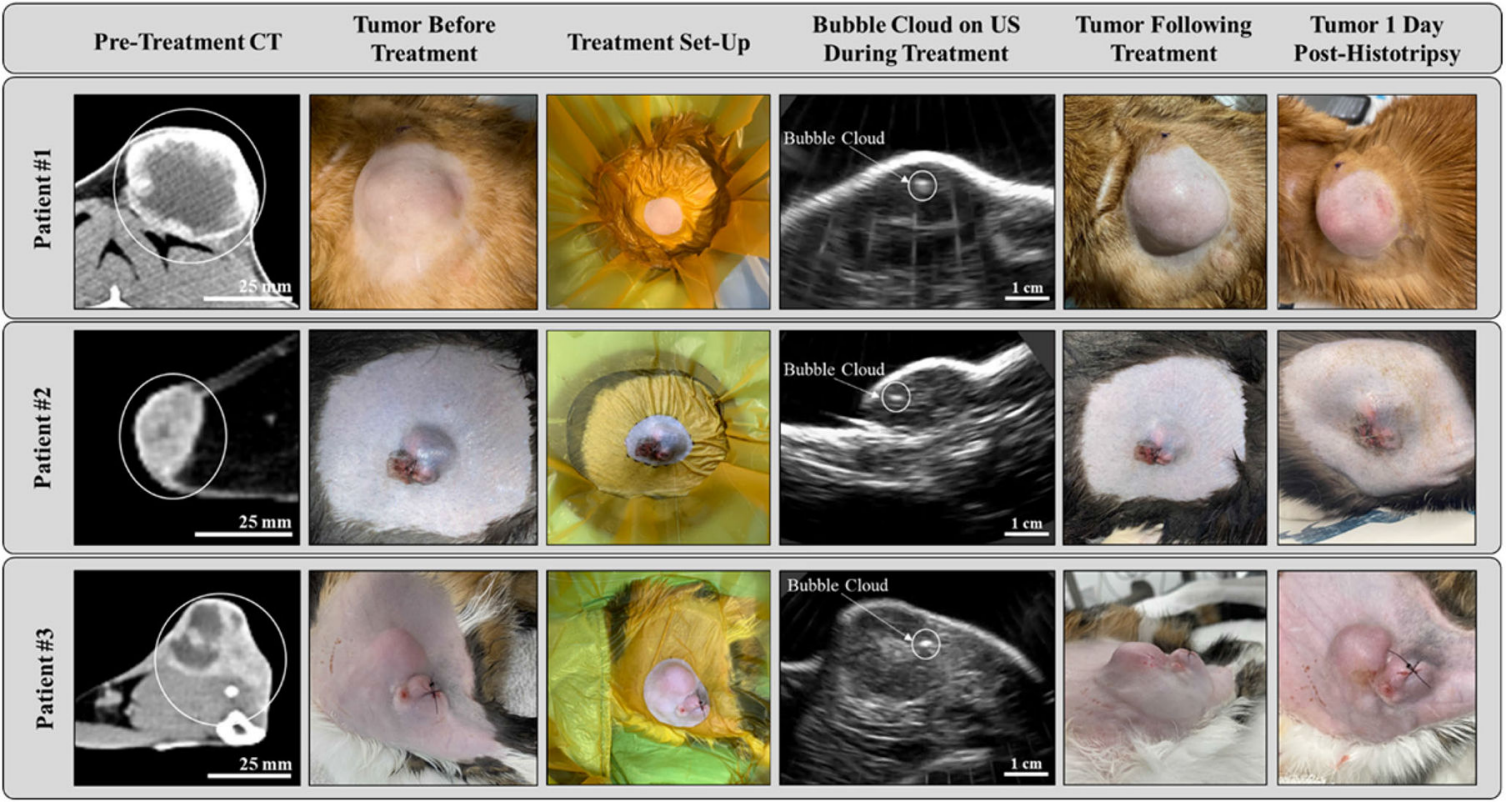
Representative images from the three feline patients show (from left to right) pretreatment CT scans revealing clearly demarcated soft tissue tumors (circled), pretreatment tumors after hair removal, water coupling baths positioned over the patient, bubble cloud formation during treatment visible on real-time US imaging, and various degrees of cutaneous injury in the histotripsy treatment path after treatment. Sutures in the skin images are from pretreatment tumor biopsies.

Patient-specific treatment plans were developed using pretreatment CT images, physical palpitation of the tumors, and freehand ultrasound imaging. Patients were anesthetized following standard protocols for client-owned cats, and anesthesia was maintained using inhaled isoflurane. Anesthesia parameters were measured every five minutes (blood pressure, pulse, and ventilation) or every fifteen minutes (oxygen saturation, carbon dioxide saturation, body temperature, and cardiac arrhythmias). To remove fur overlying the treatment area, fur was clipped and closely shaved using a razor (*n* = 1) or removed using Nair body cream (Nair Body Cream, Naircare, Ewing, NJ, USA) applied for 5–10 min (*n* = 2). Then, the histotripsy transducer was positioned over the treatment site and placed in a container of degassed water (<30% dissolved O_2_) coupled to the feline patient to ensure acoustic propagation from the transducer to the skin (Figure 3, column 3). Fine adjustments to correctly position the transducer over the targeted tumor region were made using the robotic positioner.

A portion of each tumor was treated with histotripsy according to the patient-specific treatment plan using single-cycle pulses applied at a pulse repetition frequency (PRF) of 500 Hz. To determine the appropriate pressure level for treatment, the pressure at the focus was incrementally increased until a visible bubble cloud was generated on ultrasound imaging. Then, an elliptical treatment volume fully contained within the tumor was set manually using a custom MATLAB code (The MathWorks, Natick, MA, USA), and an automated volumetric treatment was applied to a 3-D grid of equidistant treatment points within the defined boundaries. Treatment points were spaced by 1.25 mm in the axial direction and 0.75 mm in the transverse and elevational directions to allow overlap between the bubble cloud at each location. The robotic positioner moved the focus between treatment locations, and each point was treated with 500 pulses. The histotripsy treatment parameters employed in this study were chosen based on our prior study investigating histotripsy for canine STS [21,22]. All treatments were monitored in real-time using ultrasound imaging, and tumors were surgically resected 3 to 6 days after treatment.

### Evaluation of safety

The Veterinary Cooperative Oncology Group Common Terminology Criteria for Adverse Events (VCOG-CTCAE v2) was used to report any adverse events (AEs) relating to histotripsy treatment [30]. The most relevant categories for evaluation were cardiac arrhythmia, constitutional clinical signs, dermatology/skin, and musculoskeletal/soft tissue. Severe adverse events (SAEs) were defined as any grade 4/5 toxicity.

### Evaluation of ablation effectiveness

Tumor ablation was evaluated for targeting feasibility and ablation completeness using ultrasound imaging, gross assessments of the treated tumor and overlying skin, and microscopic tissue evaluation. Ultrasound imaging was used to confirm the formation of the cavitation bubble cloud during histotripsy treatment and to monitor for echogenicity changes in the treated tissues after ablation. Following surgical resection, the tumor was sectioned, and gross photographs were obtained identifying the ablation zone. Representative sections of the pretreatment tumor (from the pretreatment biopsy), treated tumor, treatment interface, and untreated tumor were fixed in 10% formalin for ≥ 24 h and embedded in paraffin. To assess the extent of histotripsy damage to the treated tissue, a standard hematoxylin and eosin (H&E) stain was used. Untreated and treated samples were stained, evaluated, and compared by a board-certified veterinary pathology with extensive experience examining ablated tumor tissue (S.C.O.).

### Characterization of the tumor microenvironment

To characterize changes in immune cell infiltration associated with histotripsy, routine immunohistochemistry was performed on formalin-fixed, paraffin-embedded (FFPE) sections of tumor taken prior to treatment, untreated tumor sampled following histotripsy treatment, and treated tumor sampled after histotripsy treatment. Tumor-associated macrophages (TAMs) and tumor-infiltrating lymphocytes (TILs) were investigated. All immunohistochemical stains were run on a Roche Ventana Discovery Ultra Automated Stainer using a heated Tris buffer for antigen retrieval and the Ultraview Red system detection system. Tissues were stained with antibodies against IBA-1 (FujiFilm, 019-19741), CD3 (Agilent Dako, A0452), and CD79a (Santa Cruz Biotechnology, HM47). All slides were counterstained with hematoxylin.

### Evaluation of the systemic immune response

Whole blood and plasma samples were collected pre-histotripsy, one day post-histotripsy, and 3–6 days post-histotripsy for each patient, archived, and used for batched cytokine analysis. Multiplex serum cytokine analysis was performed using a commercially available feline-specific antibody-coated Bead-Based Multiplex Assay to quantify 19 cytokines in each sample (FCYTMAG-20K-PMX, Millipore Sigma, Burlington, MA). The cytokines measured included Fas, Flt-3L, GM-CSF, IFNγ, IL-1β, IL-2, IL-4, IL-6, IL-8, IL-12 (p40), IL-13, IL-18, KC, MCP-1, PDGF-B, RANTES, SCF, SDF-1, and TNFα. The assay was performed by the University of Virginia Flow Cytometry Core (RRID: SCR_017829) according to the manufacturer’s directions. Samples were randomized on the plate, incubated overnight at 4 °C, and washed using a magnetic plate washer. All standards, quality controls, and samples were analyzed in duplicate on the Luminex^®^ 200^™^ multiplexing instrument (Luminex Corp., Austin, TX). Then, sample analyte concentrations were generated using standard curve data from each run *via* Belysa^®^ curve fitting software (Millipore Sigma, Burlington, MA).

### Statistical analysis

The cytokine analysis results were evaluated for normality using the D’Agostino’s K-squared test. Then, collected data for inflammatory cytokine serum concentrations were normalized *via* a log_10_ transformation and analyzed for significance using a repeated-measures analysis of variance (ANOVA). All p-values were 2-sided, and *p*-values < 0.05 were considered to be statistically significant. Analytes pre- and/or post-treatment that were lower than the minimum detection limit of the assay were excluded from statistical testing. All statistical analyses were performed using MedCalc^®^ v20.214 (MedCalc Software Ltd, Ostend, Belgium).

## Results

### Patient population and clinical outcomes

Three cats with suspected injection site sarcomas were enrolled in this study. Two female spayed cats and one male neutered cat aged 4–8 years (median: 4 years) received histotripsy treatment 3 to 6 days before surgical excision of the tumor. The characteristics of the feline patients, including the grade, subtype, and location of the tumor, are summarized in Table 1.

In all cats, the tumor was well visualized on the pretreatment CT scan and measured at least 2 cm in diameter in the longest dimension (Figure 3, column 1; Table 1). The histologic diagnosis of the tumors included 1 grade I STS (Patient #1) and 2 grade III STS (Patients #2 and #3), all consistent in location with a past injection site. Tumor sub-types included fibrosarcoma (*n* = 2; Patients #1 and #2) and myxosarcoma (*n* = 1; Patient #3). After histotripsy treatment, two cats (Patients #1 and #3) had their tumors completely excised with estimated 0.2 — 1cm and 1— 2.5 cm margins deep and laterally, respectively. In the third patient (Patient #2), three of the four lateral margins and the deep margin were clear of neoplastic cells with margins of >1.5 cm (lateral) or 0.5 cm (deep). The fourth lateral margin exhibited a questionable mix of cell types expanding into the adipose deep to the skeletal mass and extending past the surgical margin. Close monitoring for recurrence was recommended to the owner and primary care veterinarian.

At the time of manuscript preparation, two of the three cats were still alive (Patients #1 and #3). Patient outcome was followed for a median of 358 days (range: 221 – 416 days). One cat (Patient #2) was euthanized, and none were lost to follow-up. The cat that was euthanized had evidence of local recurrence at the 6-month recheck, definitively diagnosed on cytology. The owners elected for palliative care, and the cat was euthanized 8 months later. Tumor resection details and follow-up status are reported for each patient in Table 2.

### Histotripsy treatment outcomes

Histotripsy treatment was completed in three cats with suspected injection site sarcomas. Automated histotripsy treatments were applied to an ellipsoidal volume within each of the targeted tumors at peak negative pressures (*p*−) averaging 29.59 ± 6.08 MPa. Ablation volumes (Table 3) were chosen according to the size of the tumor and to minimize the time spent under anesthesia (Table 3), ranging from 0.2356 cm^3^ to 1.4726 cm^3^ (estimated); corresponding treatment times were ~18 min and 60 min, respectively (Table 3). Targeted treatment volumes correspond with 6.80 ± 4.92% (range: 3.57% — 12.46%) of the total tumor volume as determined by multiplanar reformation images from pretreatment CT scans. Treated depths ranged from 0.2 cm to 1.5 cm below the skin’s surface.

Generation of clearly visible cavitation bubble clouds on real-time ultrasound imaging was achieved in all three treatments (Figure 3, column 4). In two of three treatments (Patients #1 and #3), bubble cloud visibility was maintained throughout the length of the treatment; in the third patient (Patient #2), bubble cloud visibility was sometimes lost during treatment, and cavitation activity was monitored using passive cavitation detection (PCD) as described for previous histotripsy studies [10,22,31,32]. Patient #2’s tumor was located on the right flank, and respiratory motion impacting the tumor’s location relative to the histotripsy transducer was noted during treatment, possibly impacting the acoustic window available for histotripsy treatment. No significant changes in the echogenicity of the treated regions were noted on ultrasound imaging after treatment in any of the patients. In all three patients, intermittent-to-sustained pre-focal cavitation at the skin’s surface was observed on ultrasound imaging and/or PCD. Histotripsy treatment details are summarized by the patient in Table 3.

### Adverse event assessment

No significant histotripsy-associated AEs impacting patient outcomes were noted in any of the patients. In all patients, histotripsy was well tolerated, and no clinically significant variations from expected vital values were recorded during anesthesia. During treatment, body temperatures were maintained between 97.5 — 102.5 °F, pulse rates were maintained between 80 – 230 beats per minute, mean blood pressures were maintained between 50 – 125 mmHg, oxygen saturation levels were maintained between 97 – 100%, and end tidal carbon dioxide levels were maintained between 23 – 48 mmHg. No complications were reported during anesthetic recovery in any of the patients.

Additionally, no clinically significant AEs due to histotripsy treatment were reported through bloodwork, post-treatment physical examination, or clients’ reports. On post-treatment physical examination, no significant tumor swelling was noted for any of the patients, and the tumor was reported to be warm to the touch for one cat (Patient #3). In all three cats, mild-to-moderate ulceration of the skin was noted in the treatment path, improving during the study follow-up period before tumor resection (Figure 3, columns 5/6). None of the patients had an increase in VCOG dermatology AE score after histotripsy. One patient (Patient #3) had a VCOG post-operative wound infection AE score of 3 at the biopsy site (i.e., away from the histotripsy treatment site). Interestingly, Patient #3 had their pretreatment biopsy performed on the morning of histotripsy treatment, while Patients #1 and #2 had their biopsies performed five days pre-histotripsy. Patient #3 was admitted to the Emergency Service two days post-histotripsy for inappetence, fever, and trouble swallowing. On physical examination, purulent discharge was noted from the wound. The patient’s clinical signs resolved after receiving fluids overnight. However, this patient had a history of inappetence prior to histotripsy treatment. Amputation of the leg was performed the following day to relieve the patient’s clinical signs, and the change in the surgical timeline was not attributed to histotripsy treatment. Patient #3 had no evidence of recurrence at the 4-month recheck, and owner reports state that the patient is doing well 6-months post-histotripsy treatment.

### Gross and histologic findings

All samples were evaluated grossly at the time of submission as well as microscopically following routine processing. Treatment areas were identified *via* clinician localization at the time of sectioning. Grossly, all tumors were composed of soft to firm, white, lobulated tissue that exhibited varying degrees of tissue infiltration. In treated areas, all three samples exhibited varying degrees of tissue softening, tissue loss, and hemorrhage. In 1/3 patients (Patient #2), the ablation zone was sharply demarcated from adjacent untreated tissue; in 2/3 (Patients #1 and #3), it was less well demarcated. Two of the three samples were diagnosed as fibrosarcomas (Patients #1 and #2), and one was diagnosed as a myxosarcoma (Patient #3). Two tumors were grade III (Patients #2 and #3), and one was a grade I tumor (Patient #1) [33].

**Figure 4.**
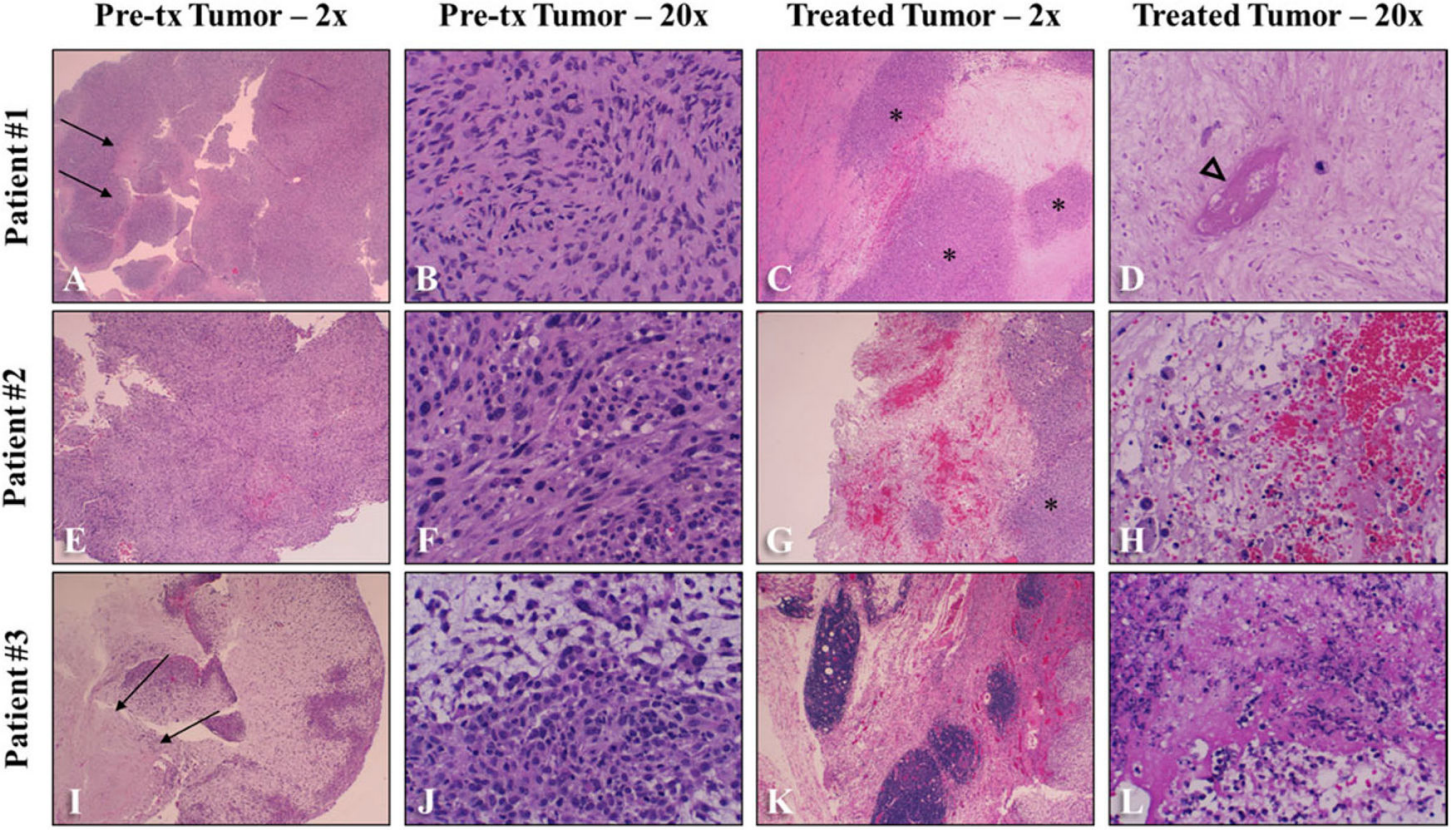
Patient-matched low- (A, C, E, G, I, K) and high-magnification (B, D, F, H, J, L) histology images comparing pretreatment (tx) and treated STS tumor samples. (A-B, E-F, I-J) Prior to histotripsy treatment, all tumors were microscopically characterized by densely cellular regions of spindle cells on a variably collagenous stroma (A-B, E-F, I-J). Occasional regions of coagulative necrosis and hemorrhage were observed in pretreatment tissues (arrows). Following histotripsy, treated tissues were characterized by large foci of coagulative necrosis (D) and/or scattered lytic necrosis (H, L), acute hemorrhage (G-H), and rare necrosis of vessels (D) (arrowhead). Intact tumor cells remained in all treated sections (asterisks). Image magnifications are reported in the figure headings.

Samples of tumor tissue collected immediately prior to histotripsy treatment (pretreatment sample), tumor tissues from the histotripsy-treated region (treated sample), and tumor tissue from regions away from the treatment site (untreated sample) were evaluated microscopically. No major differences were seen in tumor composition between pretreatment and untreated samples from the same patient. All treated areas exhibited similar features in varying amounts (Figure 4 (C-D), (G-H), (K-L)). All treatment sites exhibited foci of acute hemorrhage as well as varying degrees of acute cell death. Overall, coagulative necrosis was most prominent in treatment sites and characterized by preservation of cell architecture with cells exhibiting increased eosinophilia (pinkness), angular cell borders, and variably condensed nuclei (Figure 4(D)). Two of the three samples (Patients #1 and #3) also exhibited lytic necrosis, which was characterized by a loss of recognizable cell architecture and replacement by cellular debris (Figure 4(H, L)). Interestingly, 2/3 samples (Patients #1 and #3) also exhibited small, random foci of mostly coagulative necrosis in pretreatment and/or untreated tumor samples (Figure 4(A, I)), likely caused by standard tumor hypoxia. These foci tended to be much smaller and were not associated with the degree of acute hemorrhage seen in the treated samples. Additionally, all samples exhibited foci of intact tumor cells within the treatment site (Figure 4(C, G, K)). In 2/3 samples (Patients #2 and #3), intact tumor cells tended to be found at the periphery of the treatment zone. In 1/3 samples (Patient #1), islands of intact tumor cells were identified within the treatment zone and often centered near intact vessels. Finally, all 3 patients exhibited necrosis of few vessels within the treatment zone (Figure 4(D)).

In all three patients, grossly visible areas of ulceration were noted on the skin overlying the treatment site following histotripsy. In Patients #2 and #3, the observed ulceration correlated with microscopic abnormalities identified histologically within the skin, including necrosis, edema, and hemorrhage in the epidermis and/or the superficial dermis.

### Immunohistochemical findings

**Figure 5.**
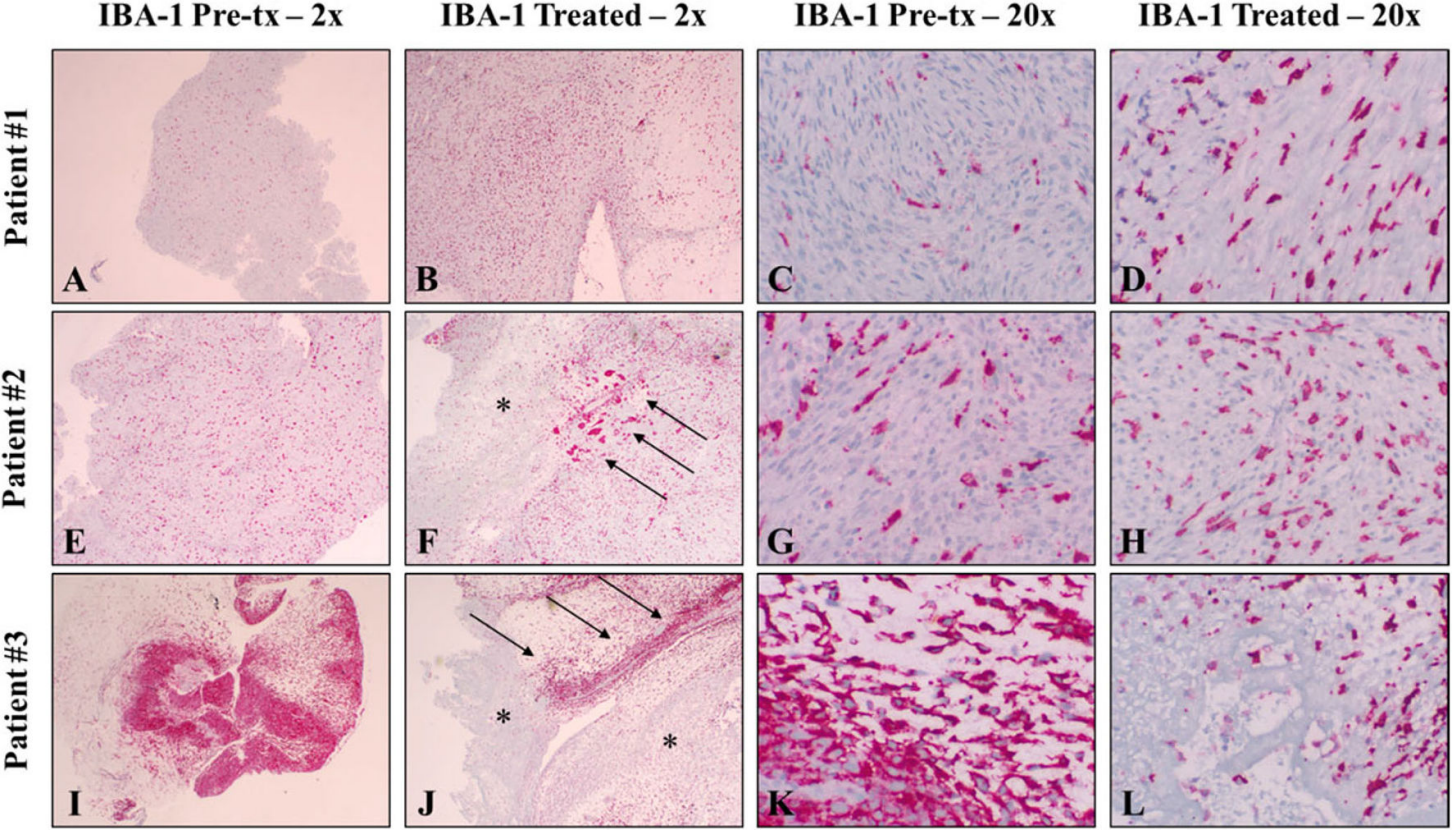
Immunohistochemistry to investigate tumor-associated macrophages (TAMs) was performed on pretreatment (tx) samples as well as untreated and treated tumor samples using antibodies again IBA-1. All samples exhibited variable numbers of readily identifiable IBA-1 positive cells in pretreatment samples (first and third column) as well as untreated tumor samples (not shown). In treated samples, 2/3 patients exhibited prominent IBA-1 positive cells at the treatment interface (arrows) as well as low to medium numbers of IBA-1 positive cells within areas of cell death (asterisks). All IHC samples utilized a red chromogen and hematoxylin counterstain. Magnifications are noted in the figure headings.

**Figure 6.**
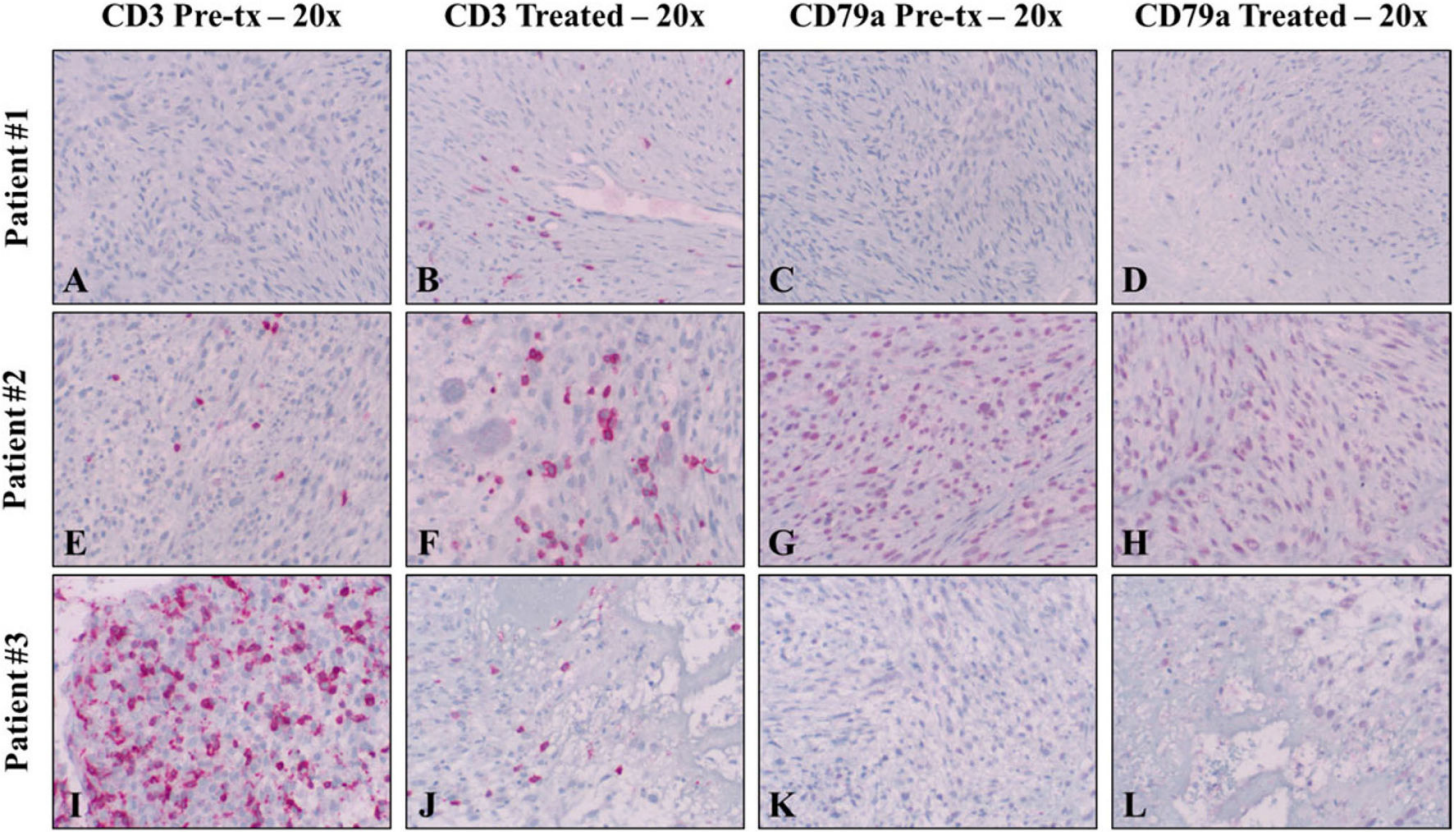
Immunohistochemistry to investigate tumor-infiltrating lymphocytes (TILs) was performed on pretreatment (tx) samples as well as untreated and treated tumor samples using antibodies against CD3 (T-cells) and CD79a (B-cells). No significant changes in TIL populations were observed between pretreatment, untreated, and histotripsy-treated samples for either antibody. All IHC samples utilized a red chromogen and hematoxylin counterstain. Magnifications are noted in the figure headings.

To investigate changes in immune cell populations associated with histotripsy treatment, immunohistochemistry was performed for IBA-1 (pan macrophage marker), CD3 (T-cell marker), and CD79a (B-cell marker) on pretreatment samples and samples from the treated and untreated tumor following histotripsy treatment. All patients exhibited readily identifiable IBA-1 positive cells throughout tumor cells both before and after treatment (Figure 5). In pretreatment and untreated samples, low numbers of sporadic IBA-1 positive cells were present in necrotic regions. In 2/3 treated tumor samples (Patients #2 and #3), IBA-1 positive cells were mildly-to-moderately dense at the treatment interface, and low-to-medium numbers of IBA-1 positive cells were located within foci of necrosis (Figure 5(F, H, J, L)). Lymphocyte analysis identified no significantly observable differences in CD3 positive cells amongst treated, untreated, or pretreatment samples; overall, total numbers of CD3 positive cells were lower than IBA-1 positive cells (Figure 6(A-B), (E-F), (I-J)). There were also no observable differences in CD79a positive cells amongst treated, untreated or pretreatment samples. Overall, no CD79a positive cells were observed amongst tumor cells in any sample (Figure 6(C-D), (G-H), (K-L)). In all tumor samples taken following treatment (treated and untreated), prominent but variably organized lymphoid follicles were present in the dermis adjacent to the mass. Lymphoid follicles were a combination of CD3 and CD79a positive cells, but composition did not appear to change between treated and untreated tumor samples at the sampled time point (not shown). No lymphoid follicles were observed in the pretreatment samples for any of the patients. The small size of the pretreatment samples, however, limited the amount of tissue available for this analysis, and it is likely that the follicles were also present prior to treatment. Immunohistochemical findings are summarized by patient in Table 2.

### Serum cytokine analysis

**Figure 7.**
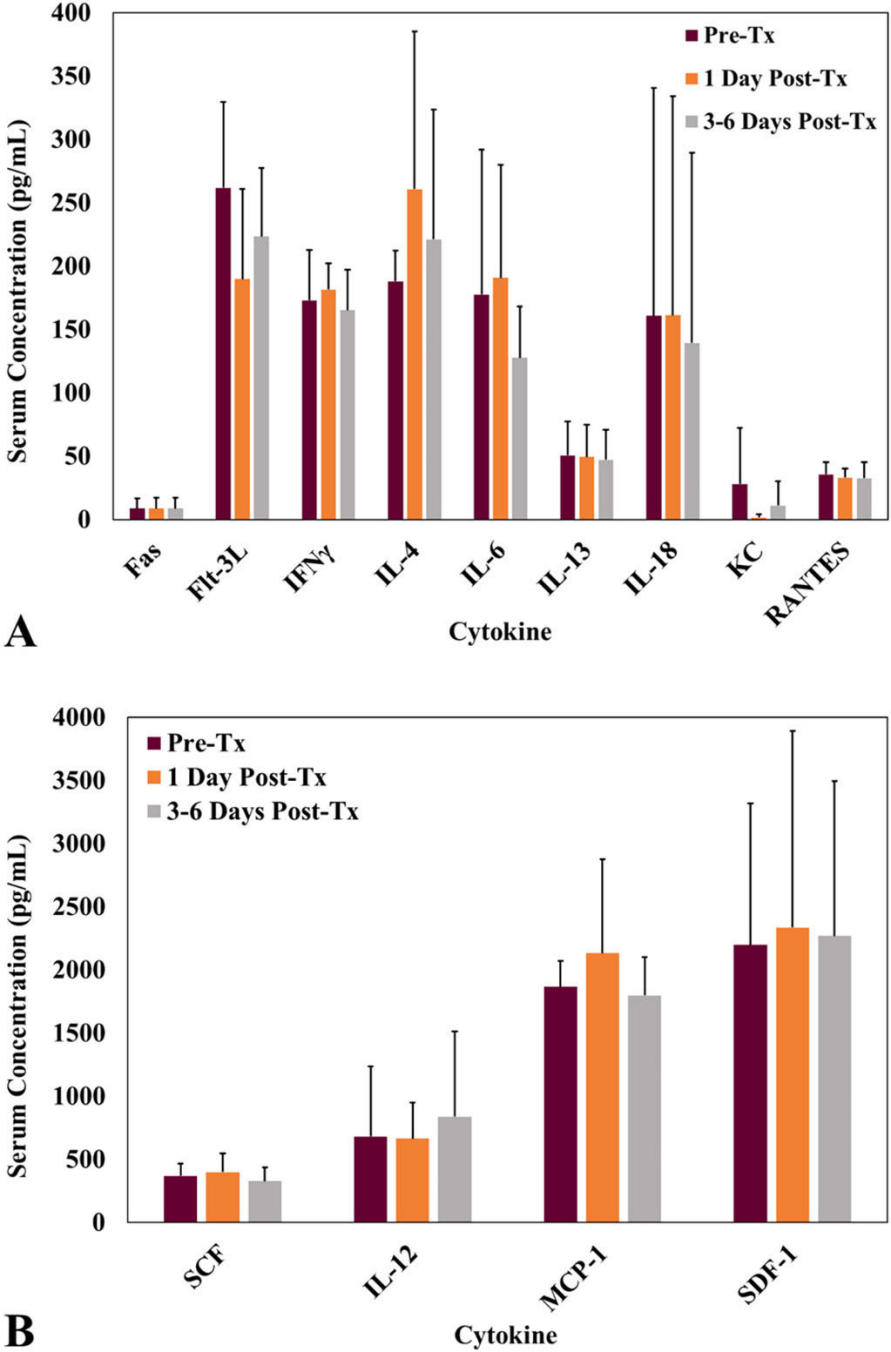
Quantified serum cytokine concentration comparison pre-, 1 day post-, and 3-6 days post-histotripsy treatment (tx) for 13 of 19 analytes. (A) Average serum concentrations for analytes Fas, Flt-3L, IFNγ, IL-4, IL-6, IL-13, IL-18, KC, and RANTES. (B) Average serum concentrations for SCF, IL-12, MCP-1, and SDF-1. *N* = 3 for all analytes. The analytes below the minimum detection limit of the assay were excluded from the figure.

Serum cytokine concentrations were compared pretreatment, one day post histotripsy treatment, and prior to surgery. Average serum cytokine concentrations ranged from 0 pg/mL (Fas, KC, and IL-18) to 3,008 pg/mL (SDF-1) prior to histotripsy treatment, 0 pg/mL (Fas, KC, and IL-18) to 3,274 pg/mL (SDF-1) one day post treatment, and 0 pg/mL (Fas, KC, and IL-18) to 3,412 pg/mL (SDF-1) three to six days post treatment. The average serum cytokine concentrations for individual patients were widely distributed for all analytes in pre- and post-treatment settings, and there were no statistically significant changes in the average patient serum cytokine concentrations following histotripsy treatment for any of the analytes (Table 4, Figure 7). Analytes GM-CSF, IL-1β, IL-2, PDGF-B, IL-8, and TNF-α were not detectable in any of the patients at all timepoints and were excluded from statistical analysis.

## Discussion

This pilot study provides initial evidence for the safety and feasibility of histotripsy for treating spontaneous feline tumors. Three cats with suspected injection site sarcomas underwent partial histotripsy ablations of their soft tissue tumors, with surgical resection of the tumors 3 to 6 days following histotripsy. In all patients, histotripsy treatment was delivered successfully, treatments were well-tolerated, and ablation of the treated regions was observed grossly and microscopically. No significant adverse events associated with histotripsy were reported for any of the patients, but mild-to-moderate skin ulceration was noted in the treatment path for all patients without an increase in VCOG dermatology AE score.

Histotripsy bubble clouds were successfully generated in the targeted tumor regions for all three patients and visibly maintained throughout the length of treatment for two patients. As noted previously, ablation of superficial tumors, including STS or fISS, poses additional challenges for histotripsy treatment due to the close proximity of the skin to the tumor body [21,22]. Pre-focal or off-target cavitation at the skin surface could cause damage to the skin, similar to the skin burns observed following superficial thermal HIFU treatments [19]. To minimize this risk, single cycle histotripsy (i.e., intrinsic threshold histotripsy) was used in this study. Single cycle histotripsy has been shown to produce a more predictable, dense bubble cloud than multi-cycle histotripsy and was previously safely employed in our canine STS histotripsy study, with only one patient of ten experiencing significant skin damage [21,22]. In the current study, mild-to-moderate ulceration of the skin was observed for all three feline patients and corresponded to the degree of pre-focal cavitation observed on real-time ultrasound imaging during treatment. Microscopic findings supported this finding of histotripsy-induced skin damage in two of the three patients. Possible causes for this include scarring and/or sutures from pretreatment biopsies within or near the treatment path or incomplete hair removal. Notably, the skin damage improved in all three patients during the follow-up period and did not require intervention or affect patient outcome.

Ablation efficacy was measured in this study using gross and histological analyses. Regions of ablation were identified grossly and histologically in the targeted regions of the resected tumors from all patients following histotripsy treatment. Histologic analysis identified large but often incomplete regions of coagulative necrosis with smaller regions of lytic necrosis and hemorrhage interspersed. Varying numbers of intact tumor cells were observed in treated tissues, with more intact and/or viable neoplastic cells remaining following treatment as compared to our previous canine STS histotripsy study [21,22]. In both studies, a treatment dosage of 500 pulses per treatment point and a pulse repetition frequency of 500 Hz were used. In this study, a custom 16-element, 1 MHz histotripsy transducer was used for all treatments instead of the 32-element, 500 kHz histotripsy transducer utilized for the canine STS histotripsy study [21,22]. This transducer was chosen due to its smaller size, shorter focal length, and higher frequency. Previous work has demonstrated that bubble cloud size decreases with increasing frequency (roughly half the size at 1 MHz vs. 500 kHz) [34], allowing for more precise targeting and finer control over the achieved ablation margins; this is especially helpful for the treatment of small, superficial tumors such as fISS. Conversely, previous studies have also demonstrated that the histotripsy ablation efficiency decreases with increasing frequency, requiring more pulses per treatment location to fully ablate the tissue at higher frequencies [34]. The latter may be the cause behind the remaining viable cells observed after treatment in the current (feline) study. In the [34] study, the average histotripsy dosage required to achieve > 75% ablation in a tissue-mimicking red blood cell phantom more than doubled at a frequency of 1 MHz compared to 500 kHz (200.7 ± 115.9 pulses vs. 74.7 ± 61 pulses, respectively). In this case, the histotripsy dose chosen for the feline study was likely underpowered and should be increased in future studies to achieve complete ablation of the entire targeted tumor volume. Another factor that may have contributed to the increased number of remaining cells is the increased f-number of the 1 MHz transducer. Past histotripsy experiments have demonstrated that the tissue fractionation efficiency decreases at higher f-numbers [29]. The elevational and transverse f-numbers for the 1 MHz transducer used herein are 0.87 and 0.70, respectively, compared to the elevational and transverse f-numbers of 0.70 and 0.61 for the 500 kHz transducer used in the canine STS study. In the referenced study [29], a hemispherical transducer was used with f-numbers ranging from 0.50 to 0.89. The number of pulses required to fractionate 50% of the treatment zone was increased from 37.5 ± 19.9 pulses at an f-number of 0.58 to 209.3 ± 17.4 pulses for an f-number of 0.89. To address this inefficiency, future studies should consider optimized devices and treatment parameters for the ablation of shallow feline tumors.

Acute immunological findings following histotripsy treatment were measured using routine H&E evaluation, IHC, and batched cytokine analysis. Microscopic evaluation demonstrated necrosis in the treated tissues and mild changes in the immune cell population after treatment, while cytokine analysis revealed no significant differences in serum cytokine concentrations up to three to six days post-histotripsy, likely due to the small sample size and/or time differences between post-histotripsy blood draws. IHC showed subjective increases in IBA-1 positive cells morphologically and immunohistochemically consistent with macrophages in treated tumor sections, echoing macrophage population findings in both the thermal and mechanical HIFU canine STS studies [19,21,22]. Occasionally, IBA-1 positive cells were also identified in necrotic regions of pretreatment and untreated tumor samples, but were lower in number than treated tumor sections, suggesting that the cell death at the histotripsy treatment sites may be more immunogenic than cell death secondary to tumor hypoxia alone. No significant changes in the number or distribution of CD3 or CD79 positive cells were noted between samples in this study, which suggests that few lymphocytes were recruited to the treatment site following histotripsy. Interestingly, a recent study investigating histotripsy’s impact on metastatic development in a rodent liver tumor model identified increased infiltration of CD11b positive cells, CD8 positive T-cells, and natural killer (NK) cells at the histotripsy ablation zone seven days following treatment when > 50% of the tumor was ablated, but not when < 25% of the tumor was targeted [35]. Likewise, another study reported an association between histotripsy treatment and abscopal, intratumoral CD8 positive T-cell responses in distant tumors at 10 days post-histotripsy [24]. In this study, however, targeted treatment volumes ranged from 3 – 12% of the total tumor volume, and treated tissues were surgically removed 3 to 6 days after histotripsy. As a result, it is possible that stronger immune trends may be identified following histotripsy ablation of increased volumes within patient tumors or at later timepoints. Future studies with more cats should investigate immune changes in feline STS following histotripsy treatments of > 50% of the tumor volume as well as consider additional timepoints and markers for immune cell subtypes.

Overall, the results of this study demonstrate, for the first time, that histotripsy has the potential to be used as a non-invasive ablation therapy for feline cancers, specifically feline STS and injection site sarcoma. While the current study is limited by its small patient population, its findings invariably support additional studies to fully characterize histotripsy’s role as an ablative and possibly immunostimulatory therapy for feline malignancies. In this first-in-feline study, treated tumors were surgically removed after histotripsy treatment to definitively treat the cats for their cancer and to allow for gross and histological analyses of the treated volumes. Future studies should investigate whether histotripsy can be used to fully ablate complete feline STS or fISS tumors with adequate margins to prevent local recurrence while sparing the skin as well as the role of post-histotripsy imaging to characterize the achieved ablation in cats. In the current study, incomplete ablation of the targeted region was achieved, likely due to an inadequate histotripsy dose being applied; as a result, future studies should explore the required histotripsy dose to fully emulsify the target tissue for feline STS. Further, future work remains to develop the optimal histotripsy device for this application. For example, the limitations of the 1 MHz device discussed above (i.e., lower ablation efficiency, longer treatment time for large tumors) suggest that there may be an intermediate transducer frequency, such as 750 kHz, which would be better suited to this application, balancing precision while minimizing the required treatment time. Likewise, the use of a more focused histotripsy transducer with a lower f-number may further increase the ablation efficiency. Finally, future studies should consider methods to more extensively characterize the acute and chronic immune responses following partial and complete STS tumor ablation in cats.

Different clinical pathways for histotripsy of feline STS should also be explored. Based on the needs of the patient, histotripsy could be implemented clinically as either a primary or adjunctive therapy. In cases where complete surgical resection is impossible, histotripsy could be used as the primary treatment option for local tumor control; alternatively, histotripsy could be applied as a neoadjuvant therapy to target the leading edge of the tumor, decreasing the required surgical margins, and/or to promote an abscopal effect. In instances of tumor recurrence, histotripsy could serve as an alternative local therapy when retreatment with conventional therapies is infeasible (e.g., maximal radiation dose has been reached or surgery is impossible). A combination of these approaches should also be considered.

## Conclusion

Histotripsy is a safe and feasible noninvasive therapy for feline injection site sarcomas. While work remains to fully develop histotripsy for this application, the results of this first-in-feline study show that histotripsy treatment was well-tolerated and accurate in feline patients with STS and guide the clinical development of histotripsy devices for this purpose. As expected, acute immunological results suggest that histotripsy was able to induce pro-immunogenic changes in the local tumor microenvironment, and future work should consider the chronic immune response after histotripsy. Overall, this work suggests that histotripsy is a promising therapy for the noninvasive treatment of feline STS, and future trials investigating histotripsy for the complete ablation of feline STS and fISS as well as for other feline cancers are warranted.

## Figures and Tables

**Table 1.** Characteristics of the feline patients and soft tissue tumors. Patients are ordered by the date of histotripsy treatment, and tumor sizes are reported as the maximal diameter in each direction based on multiplanar reformation images from pretreatment CT scans. Abbreviations: MN: male neutered, FS: female spayed, PD: primary diagnosis; ST: subtype.

Patient #	Age (years)	Gender	Tumor Details	Tumor Location	Tumor Size (cm)
1	4	MN	*PD:* Grade I STS *ST:* Fibrosarcoma	Left dorsal shoulder	3.2 × 3.8 × 4.1
2	4	FS	*PD:* Grade III STS *ST:* Fibrosarcoma	Right flank	1.8 × 2.5 × 2.9
3	8	FS	*PD:* Grade III STS *ST:* Myxosarcoma	Left proximal hind limb	*Lobe 1:* 2.4 × 2.4 × 2.7 *Lobe 2:* 1.8 × 2.0 × 2.1

**Table 2.** Clinical and immunological outcomes organized by patient.

Patient #	Excision Type	Tumor Recurrence	Follow-up Status	Immune Response
1	Marginal; complete	No	Alive	No clear immunohistochemical trends
2	Marginal; incomplete	Yes	Euthanized	Mild-to-moderate IBA-1 positive staining at the treatment interface; low-to-medium numbers of IBA-1 positive cells in necrotic foci
3	Amputation; complete	No	Alive	Mild-to-moderate IBA-1 positive staining at the treatment interface; low-to-medium numbers of IBA-1 positive cells in necrotic foci

**Table 3.** Histotripsy treatment details summarized by patient. *The treatment duration for Patient #1 was not recorded.

Patient #	Targeted Volume (cm)	Treatment Pressure (MPa)	Treatment Depth (cm)	Bubble Cloud Visibility	Pre-focal Cavitation	Treatment Duration (min)
1	1.0 × 1.0 × 2.2	35.3	1.0	Yes	Intermittent	N/A*
2	0.6 × 0.75 × 1.0	23.2	0.50	Intermittent	Yes	17.88
3	1.25 × 1.5 × 1.5	30.3	0.75	Yes	Yes	59.73

**Table 4.** Summary of the serum cytokine concentrations pretreatment (tx), 1-day post-treatment, and 3–6 days post-treatment. No statistically significant differences were observed. The analytes below the minimum detection limit of the assay were excluded from the table.

Cytokine	Pre-Tx		1 Day Post-Tx		3–6 Days Post-Tx	
	Median (pg/mL)	Range (pg/mL)	Median (pg/mL)	Range (pg/mL)	Median (pg/mL)	Range (pg/mL)
*Fas*	11.99	0–14.59	10.74	0–15.92	10.74	0–15.92
*Flt-3L*	253.78	198.60–333.29	157.46	140.87–271.27	219.13	171.98–279.58
*IFNγ*	153.39	146.35–218.92	188.25	158.46–198.39	163.36	134.43–198.39
*IL-4*	187.90	164.17–212.54	251.03	141.44–389.89	200.11	130.50–332.65
*IL-6*	145.98	82.47–304.57	157.35	128.82–291.75	140.36	82.47–160.22
*IL-13*	51.48	23.72–77.07	54.59	21.73–72.19	50.21	21.73–69.40
*IL-18*	127.23	0–355.09	140.38	0–343.52	120.76	0–297.77
*KC*	5.34	0–78.89	0	0–4.82	0	0–32.98
*RANTES*	37.19	25.50–44.28	36.02	24.93–38.37	27.82	23.20–47.24
*SCF*	332.85	293.35–482.33	345.17	290.88–567.22	268.62	261.19–454.30
*IL-12*	379.71	342.69–1322	543.43	463.67–990.35	655.90	270.89–1585
*MCP-1*	1935	1635–2032	1837	1583–2977	1737	1531–2126
*SDF-1*	2672	919.54–3008	3193	541.97–3274	2420	975.14–3412

## Data Availability

The data presented in this study are available on request from the corresponding authors.
